# danRerLib: a Python package for zebrafish transcriptomics

**DOI:** 10.1093/bioadv/vbae065

**Published:** 2024-05-06

**Authors:** Ashley V Schwartz, Karilyn E Sant, Uduak Z George

**Affiliations:** Computational Science Research Center, College of Sciences, San Diego State University, San Diego, CA 92182, United States; Computational Science Research Center, College of Sciences, San Diego State University, San Diego, CA 92182, United States; Division of Environmental Health, School of Public Health, San Diego State University, San Diego, CA 92182, United States; Computational Science Research Center, College of Sciences, San Diego State University, San Diego, CA 92182, United States; Department of Mathematics and Statistics, College of Sciences, San Diego State University, San Diego, CA 92182, United States

## Abstract

**Summary:**

Understanding the pathways and biological processes underlying differential gene expression is fundamental for characterizing gene expression changes in response to an experimental condition. Zebrafish, with a transcriptome closely mirroring that of humans, are frequently utilized as a model for human development and disease. However, a challenge arises due to the incomplete annotations of zebrafish pathways and biological processes, with more comprehensive annotations existing in humans. This incompleteness may result in biased functional enrichment findings and loss of knowledge. danRerLib, a versatile Python package for zebrafish transcriptomics researchers, overcomes this challenge and provides a suite of tools to be executed in Python including gene ID mapping, orthology mapping for the zebrafish and human taxonomy, and functional enrichment analysis utilizing the latest updated Gene Ontology (GO) and Kyoto Encyclopedia of Genes and Genomes (KEGG) databases. danRerLib enables functional enrichment analysis for GO and KEGG pathways, even when they lack direct zebrafish annotations through the orthology of human-annotated functional annotations. This approach enables researchers to extend their analysis to a wider range of pathways, elucidating additional mechanisms of interest and greater insight into experimental results.

**Availability and implementation:**

danRerLib, along with comprehensive documentation and tutorials, is freely available. The source code is available at https://github.com/sdsucomptox/danrerlib/ with associated documentation and tutorials at https://sdsucomptox.github.io/danrerlib/. The package has been developed with Python 3.9 and is available for installation on the package management systems PIP (https://pypi.org/project/danrerlib/) and Conda (https://anaconda.org/sdsu_comptox/danrerlib) with additional installation instructions on the documentation website.

## 1 Introduction

Transcriptomics is a widely used methodology for assessing gene expression changes in response to experimental conditions and plays a pivotal role in understanding the molecular mechanisms governing biological processes and disease. RNA-sequencing is the most common form of investigating differential gene expression changes that gives an unbiased snapshot of the transcriptome (Stark *et al.* 2019). The zebrafish (Danio rerio) is an established vertebrate model for human development and disease, especially in embryonic toxicity studies and developmental biology (Link and Megason 2008, Yang *et al.* 2009, Roper and Tanguay 2018). With a transcriptome that closely mimics that of humans (Howe *et al.* 2013), zebrafish offer a unique opportunity to investigate the effects of experimental conditions on gene expression.

The quantification and interpretation of gene expression changes often involve performing functional enrichment analysis to discern the under- or over-expression of specific biological processes or pathways (Huang *et al.* 2009a). For example, pharmacologists and toxicologists commonly conduct functional enrichment analyses to assess the molecular impact of exposures or treatments. Through the analysis, researchers are able to identify key up- and downregulated pathways following exposure (Navarrete *et al.* 2021, Haimbaugh *et al.* 2022, Perkins *et al.* 2022). However, existing tools frequently lack tailored support for zebrafish researchers, and updates to databases like Kyoto Encyclopedia of Genes and Genomes (KEGG) (Kanehisa *et al.* 2023) or Gene Ontology (GO) (Gene Ontology Consortium 2021) are not always readily available. An additional challenge is the fact that many pathways and biological processes, which are known to also occur in zebrafish, remain unannotated for zebrafish with more comprehensive annotations existing in humans. For instance, KEGG Current Release 109.1 lists 179 pathways for zebrafish and a total of 357 annotated for human. It is important to note that the KEGG disease database is only designed for humans, which poses a challenge for researchers who want to utilize zebrafish as a model for relevant human diseases. The current limitations of existing tools may lead to incomplete, outdated, and biased functional annotation results, which in turn may hinder the interpretation of experimental outcomes.

To address these limitations, we introduce *Danio rerio* Library, danRerLib, a specialized Python package for zebrafish researchers utilizing transcriptomics in their research. Uniquely designed to support the latest genome and annotation builds for KEGG and GO, danRerLib extends conventional gene tools by providing dedicated gene conversion and functional enrichment tools tailored explicitly for zebrafish studies. A primary contribution is the incorporation of orthology-based functional enrichment analysis, enabling the study and testing of pathways and biological processes not currently annotated for zebrafish.

## 2 Methods

**Figure 1.**
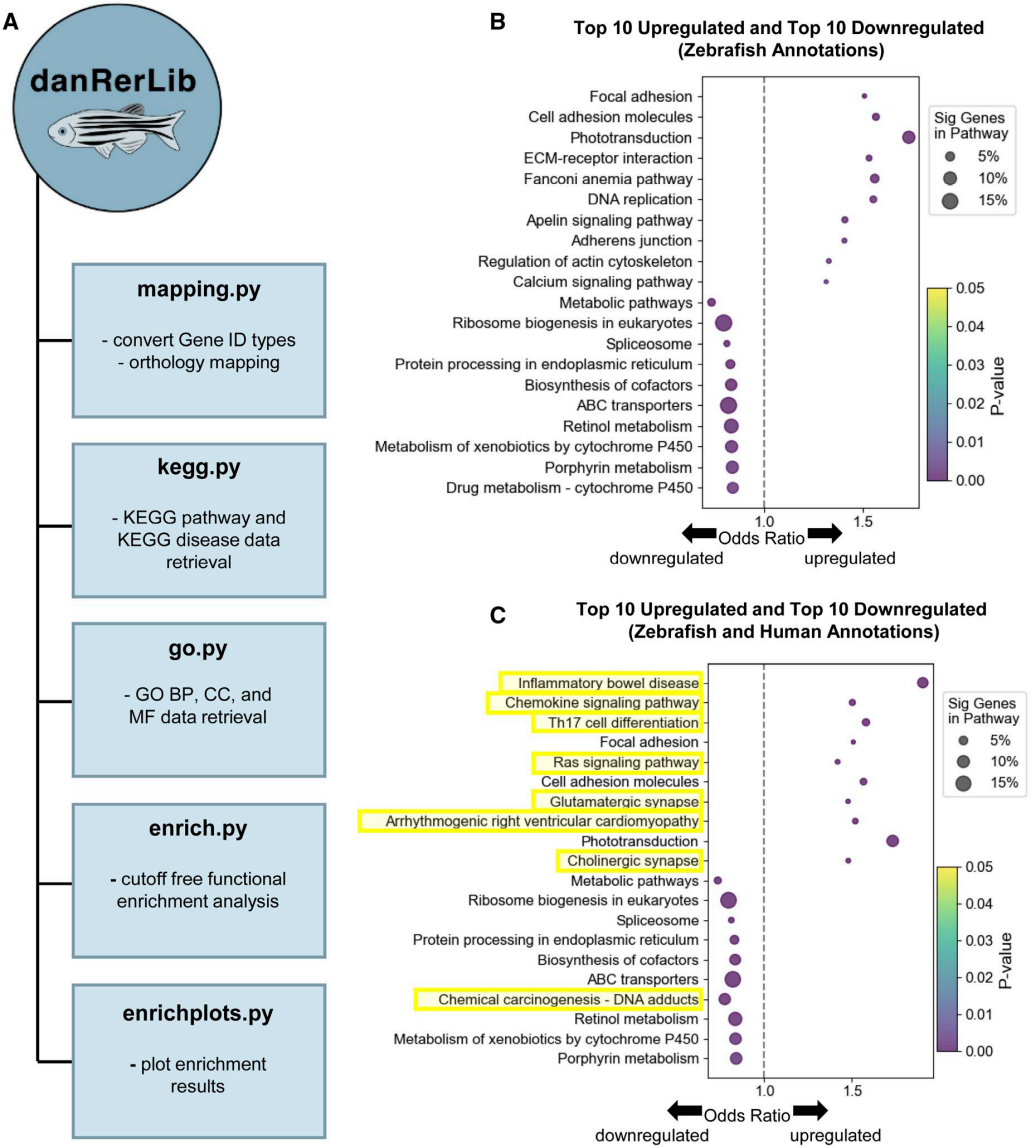
(A) danRerLib modules and key capabilities. (B) The top 10 upregulated and the top 10 downregulated KEGG pathways using zebrafish pathway annotations. (C) The top 10 upregulated and the top 10 downregulated KEGG pathways using zebrafish pathway annotations and the addition of KEGG pathways using human annotations via orthology. Novel pathways discovered via orthology and not present in (B) are highlighted in yellow. In (B) and (C), the dot color represents the significance of up/downregulation of the pathway and the dot size represents the percentage of significant genes within the pathway.

The Python package danRerLib is divided into five key modules: the mapping module, the KEGG module, the GO module, enrichment module, and the enrichplots module. The functionality and purpose of each of the included modules are described below and highlighted in Fig. 1A.

### 2.1 Mapping module

The mapping module has been designed to convert Gene IDs from different databases and gene nomenclatures, including National Center for Biotechnology Information (NCBI) Entrez Gene IDs (Maglott *et al.* 2007), Ensembl Gene IDs (Martin *et al.* 2022), Zebrafish Information Network (ZFIN) IDs (Bradford *et al.* 2022), and official Gene Symbols (Bradford *et al.* 2022). In addition, the mapping module facilitates orthology mapping between human and zebrafish taxonomy, using orthologous genes defined and managed by ZFIN (Bradford *et al.* 2022).

### 2.2 KEGG module

The KEGG module contains tools for the investigation of genes within a KEGG pathway or KEGG disease. The KEGG pathway database contains pathway maps that encapsulate our knowledge of cellular and organism-level functions, categorized into metabolism, genetic information processing, environmental information processing, cellular processes, organismal systems, and human diseases (Kanehisa *et al.* 2023). The KEGG disease database describes molecular networks caused by perturbants including gene variants, viruses and other pathogens, and various environmental factors (Kanehisa and Goto 2000). One can download a list of genes given a KEGG pathway or disease ID in any supported Gene ID format.

### 2.3 GO module

The GO module contains tools to probe information from the Gene Ontology databases including GO Biological Processes (GO BP), GO Molecular Functions (GO MF), and GO Cellular Components (GO CC). Gene Ontology categorizes gene products into structured ontologies, providing valuable insights into their biological roles, molecular activities, and cellular compositions (Ashburner *et al.* 2000). The GO module allows for the exploration of these ontologies, allowing users to query ontologies by GO ID and analyze genes based on their involvement in biological processes, molecular functions, and cellular components. One can download a list of genes given a GO ID in any supported Gene ID format.

### 2.4 Enrichment module

The enrichment module is equipped with tools designed for conducting functional enrichment analysis of gene sets within KEGG pathways, KEGG diseases, and Gene Ontology (GO) ontologies. Users gain the capability to assess the degree of enrichment for gene sets based on their datasets of gene expression, determining the overrepresentation or underrepresentation of genes within specific gene sets. The enrichment module allows for flexibility to conduct a global analysis across all gene sets or focus on specific IDs or subsets. Users will receive a detailed data table containing all IDs of gene sets significantly enriched, depleted, upregulated, or downregulated depending on whether a directional test is chosen. Two methods of functional enrichment analysis are currently supported including overrepresentation analysis by Fisher’s exact test and the logistic regression method (Sartor *et al.* 2009, Lee *et al.* 2015). The *P*-value of Fisher’s exact test is computed utilizing the hypergeometric distribution as shown in Equation 1.

(1) p=a+bac+ddNa+c .

In Equation (1), a is the number of genes in the gene set and significantly expressed, b is the number of genes not in the gene set that are significantly expressed, c is the number of genes in the gene set and nor significantly expressed, d is the number of genes neither in the gene set nor significantly expressed, and N is the total number of genes in the background. The logistic regression model can be described using Equation (2).

(2) $$\log\left(\frac{p}{1-p}\right)=\alpha +\beta x,\;$$

where the explanatory variable x is defined as the negative log of the pvalues for differential expression, $-\log\left(p-\mathrm{value}\right)$, p is the probability of a gene belonging to a gene set, α is the intercept, and β is the slope parameter. The Wald test is then utilized to assess the evidence that β is significantly different from zero following a $\chi^{2}$ distribution with one degree of freedom and is defined in Equation (3).

(3) $$W=\left(\frac{\hat{\beta }}{s_{\hat{\beta }}}\right).\;$$

The slope parameter β corresponds to the log odds of belonging to a gene set and when β>0 the gene set is considered to be enriched (Sartor *et al.* 2009).

### 2.5 Enrichplots module

The enrichplots module complements the enrichment module, providing users with powerful visualization tools to effectively interpret and communicate the results obtained from functional enrichment analyses. With the enrichplots module, users can create a variety of plots, including bar charts, volcano plots, and dot plots. Bar charts can show the distribution of enriched, depleted, upregulated, or downregulated gene sets across different categories. Volcano plots help users understand the relationship between statistical significance and fold change, offering a comprehensive view of significantly altered pathways. Dot plots portray key enrichment metrics such as the odds ratio, the significance of pathway enrichment, and the percentage of significant genes within the pathway. A dot plot example is shown in Fig. 1 with the remaining options illustrated in Supplementary Fig. S1. These visualization tools enable users to extract meaningful insights and communicate the biological relevance of significantly altered gene sets more effectively.

The databases used for the described modules have been built using the latest genome builds and nomenclature from ZFIN (release 18 March 2024), NCBI (FTP release 18 March 2024), and GO at the time of development in March 2024 from the respective databases. The KEGG data is accessed through web API to ensure the latest online data is being utilized (Release 109.1 API, 1 March 2024). Database updates will be updated quarterly. If a user wishes to update to the latest database build, each database can be rebuilt using the building functions within the modules to ensure the latest information is being utilized.

## 3 Results

A primary contribution of danRerLib is the ability to streamline functional enrichment analysis for pathways and gene sets not annotated for zebrafish through orthology. To illustrate this contribution, we analyzed our previously published RNA-sequencing data of 4-day postfertilization whole embryo zebrafish following exposure to the environmental contaminant tris(4-chlorophenyl)methanol (TCPMOH) (Navarrete *et al.* 2021). In Navarrete *et al.* (2021), we performed functional enrichment analysis using LR Path (Sartor *et al.* 2009) which relies solely on zebrafish annotations. In the current study, we performed functional enrichment analysis using danRerLib’s cut-off free logistic regression method and compared the findings using zebrafish annotations alone to those incorporating additional pathways via orthology. This approach showcases the enhanced capabilities of danRerLib in expanding the scope of functional enrichment analysis beyond zebrafish annotated pathways. A detailed comparison demonstrating the benefit of orthology and a comparison of danRerLib to the existing tools including DAVID (Huang *et al.* 2009b, Sherman *et al.* 2022), gProfiler (Kolberg *et al.* 2023), FishEnrichr (Chen *et al.* 2013, Kuleshov *et al.* 2016), GSEApy (Fang *et al.* 2022), LRPath (Sartor *et al.* 2009), clusterProfiler (Yu *et al.* 2012, Wu *et al.* 2021), and GOAtools (Klopfenstein *et al.* 2018) are included in the Supplementary Material.

We identified 55 significantly upregulated/downregulated KEGG pathways based on zebrafish annotations alone (*P* < .05). The top 10 up and top 10 downregulated pathways are shown in Fig. 1B. After expanding the analysis to include orthology, we identified a total of 95 KEGG pathways that were significantly upregulated or downregulated. This emphasizes the importance of orthology-based enrichment analysis in capturing biologically relevant information that may not be evident in zebrafish annotations alone. Out of the 55 pathways that were identified as significant using zebrafish annotations, 42 were also found to be present in the human annotation test. However, the identification of 13 significant pathways exclusively through zebrafish annotations unveils the complex relationship between human and zebrafish genomes.

The complexity between humans and zebrafish comes from the lack of one-to-one orthology, primarily due to a whole genome duplication event in teleost fish evolution. Based on the latest human genome build reported in NCBI (v.GRCh38) incorporated in the building of danRerLib, there are 20 644 human protein-coding genes and 13 508 of these genes have at least one zebrafish ortholog as reported in ZFIN; therefore, at least 65% of human genes have at least one zebrafish ortholog. This mismatch gene orthology extends to gene sets and is a significant contributor to the divergence in enrichment results using orthology-based annotations. Therefore, it is necessary to consider the relationship between species and interpret functional enrichment analyses accordingly.

To tackle the complexity of cross-species gene relationships, danRerLib offers a solution that allows users to selectively incorporate only human annotations that lack counterparts in zebrafish. This approach makes use of zebrafish annotations as the ground truth, enabling researchers to accurately interpret the significance of pathways in the context of zebrafish-specific genomic intricacies. Using this the orthology mapping in danRerLib, we identified a total of 95 significantly upregulated or downregulated pathways with the top 10 upregulated and top 10 downregulated are shown in Fig. 1C.

One of the noteworthy aspects of the methodology proposed with danRerLib is the examination of pathways that are associated with human disease, given that KEGG disease annotations are available only for humans. Using zebrafish annotations alone as shown in Fig. 1B, we discovered that KEGG metabolic pathways were considerably decreased. This result is further confirmed in Fig. 1C with the inclusion of human annotations. However, by leveraging zebrafish annotations in KEGG and incorporating orthology, we uncovered additional insights. Notably, inflammatory bowel disease emerged as a top significantly upregulated pathway. This finding via danRerLib explains the pathological observation of intestinal effusion in the microscopy images of the zebrafish in Navarrete *et al.* (2021). Thus, danRerLib provides valuable information about how TCPMOH may be linked to the underpinnings of disease. These findings using the streamlined orthology-based enrichment analysis highlight the benefit of investigating both zebrafish and human annotations, a method often overlooked and requiring extra steps in existing methods. Introducing significant pathways and diseases can assist in comprehending complex disease manifestations that occur following exposure to chemicals.

## 4 Conclusion

danRerLib provides a tailored and streamlined approach to gene ID conversions, orthology mapping, and functional enrichment analysis specifically for zebrafish researchers in Python. The incorporation of orthology enables researchers to overcome the limitations of direct zebrafish annotations, allowing for the exploration of pathways and biological processes not previously annotated. Though not all human diseases necessarily occur in zebrafish, nor are the same molecular processes necessarily conserved in disease progression between species, identification of these additional pathways can provide keen insights into pathophysiology. Given the growing popularity of Python among researchers, danRerLib provides a valuable resource for zebrafish researchers to complement bioinformatics workflows in Python while integrating multiple enrichment methodologies. Furthermore, danRerLib supports users of all computational experiences, providing comprehensive tutorials that guide users through the functionalities and maximize the package’s utility.

## Supplementary Material

vbae065_Supplementary_Data
