# Supplementary material for: danRerLib: a Python package for zebrafish transcriptomics
Source: Bioinform Adv. 2024 May 6;4(1):vbae065. doi: 10.1093/bioadv/vbae065 (PMC11105952; doi:10.1093/bioadv/vbae065)
Supplement: vbae065_Supplementary_Data [file vbae065_supplementary_data.docx]

| *Supplementary Information*  **danRerLib: a Python Package for Zebrafish Transcriptomics**  Ashley V. Schwartz^1^, Karilyn E. Sant^1,2^ and Uduak Z. George^1,3, *^  ^1^Computational Science Research Center, San Diego State University, 5500 Campanile Dr, San Diego, CA 92182, USA, ^2^School of Public Health, San Diego State University, 5500 Campanile Dr, San Diego, CA 92182, USA, ^3^Department of Mathematics and Statistics, San Diego State University, 5500 Campanile Dr, San Diego, CA 92182, USA.  *To whom correspondence should be addressed. |
| --- |

**Comparison of danRerLib to Existing Tools**

We analyzed our previously published RNA-sequencing data of 4 days post fertilization whole embryo zebrafish following exposure to the environmental contaminant tris(4-chlorophenyl)methanol (TCPMOH) (Navarrete, et al., 2021) and compared the results to existing functional enrichment tools: DAVID (Sherman, et.al, 2022; Huang, et al., 2009), gProfiler (Kolberg et. al., 2023), FishEnrichr (Chen et.al., 2013; Kuleshov et. al., 2016), GSEApy (Fang et. el., 2022), LRPath (Sartor et. al., 2009), clusterProfiler (Wu et. al., 2021; Yu et. al., 2011), and GOAtools (Klopfenstein et. al., 2018). In our comparison, we examined the features of danRerLib alongside seven other functional enrichment analysis tools, as depicted in Table S1. Through our analysis, we found three notable features that distinguish danRerLib in terms of functionality and utility. Firstly, it caters specifically to Python users in zebrafish transcriptomics, incorporating updated functional annotation databases. Secondly, it utilizes a cut-off free logistic regression method for enrichment testing. Thirdly, it incorporates orthology-based enrichment analysis.

Among the list of 8 tools in Table S1, there is an important difference in Python support. Four of them, namely gProfiler, GSEApy, GOATools, and danRerLib, have Python packages and documentation. However, only two of them are regularly updated with the latest functional annotation database builds for KEGG and GO. The updated version of gProfiler is primarily a web-based tool, but it includes a Python wrapper for the API. Although useful, gProfiler does not support zebrafish gene id types such as ZFIN, which means users must use multiple tools to complete enrichment analysis if they are using ZFIN gene ids. On the other hand, danRerLib is a tool that specifically meets the needs of zebrafish researchers who use Python in their workflows. As Python becomes increasingly popular in bioinformatics workflows, danRerLib will continue to support the community.

One of the most common methods for determining whether a group of genes is overrepresented in a gene set involves calculating the number of differentially expressed genes within the set and computing a p-value using Fisher's exact test. This approach is employed by the danRerLib enrich_fishers function in the enrichment module, as well as by other established enrichment tools such as David, gProfiler, Fishenrichr, GSEApy, clusterProfiler, and GOAtools. However, a significant limitation of this method is that it requires a threshold for significance to determine which genes are considered differentially expressed and therefore tested against the gene set. The choice of threshold can have a significant impact on the results of the enrichment analysis, as noted by Pan et al. (2005). To offer users flexibility in overcoming this limitation, danRerLib provides a cut-off-free logistic regression method for enrichment testing developed by Sator et al. (2009). To our knowledge, danRerLib is the only fully updated tool to conduct functional enrichment analysis using the logistic regression method with the latest functional annotation database builds as the web-based tool LRPath is utilizing KEGG annotations, for example, from 2011.

danRerLib introduces a hybrid orthology-based enrichment analysis approach, addressing the incompleteness of functional annotations in zebrafish compared to humans. For instance, while KEGG currently lists 179 pathways for zebrafish, it includes 347 pathways for humans. To leverage the broader scope of human annotations, danRerLib enables users to test these additional pathways while treating zebrafish annotations as the baseline. This unique feature is not offered by any of the tools listed in Table S1. Using orthology-based approaches in other tools often necessitates mapping the gene list from zebrafish to human and subsequently testing human-annotated pathways. However, this method risks overlooking zebrafish-specific nuances. In contrast, danRerLib offers a hybrid approach, automatically prioritizing zebrafish annotations when available and employing orthology to test human-annotated pathways only in the absence of zebrafish annotations. This ensures a comprehensive enrichment analysis considering both zebrafish-specific and conserved biological pathways.

To illustrate the benefit of danRerLib, we conducted functional enrichment analysis to determine enriched, depleted, upregulated, and downregulated KEGG Pathways utilizing all tools listed in Table S1 that contain KEGG support. Enriched pathways indicate overrepresentation of significant genes, while depleted pathways are underrepresented. Upregulated pathways contain overrepresented upregulated genes, whereas downregulated pathways contain overrepresented downregulated genes. The number of significantly identified pathways for each tool listed has been taken for a pvalue < 0.05 and shown Table S2. The enrichment testing options offered by danRerLib, such as fishers, logistic, fishers via orthology, and logistic via orthology, are included in the tests shown in Table S2. One significant advantage of danRerLib’s orthology enrichment is that it nearly doubles the number of KEGG pathways tested, from 179 to 357. The updated annotation database of danRerLib is beneficial for outdated tools that have fewer annotated pathways to test against. Additionally, danRerLib offers the capability to test for pathway depletion, a feature shared only by gProfiler and LR Path.

**Supplementary Tables**

**Table S1**: Features of common enrichment testing tools and danRerLib.

| Enrichment Tool | Statistical Test | Web- Based | Python Pack-age | | R Pack-age | | KEGG Knowl-edgebase Update | | GO Knowl-edgebase Update | Adj p-value Method | Orthology Tool | Orthology Based Enrich-ment Analysis | Supports ZFIN IDs for Enrich-ment |
| --- | --- | --- | --- | --- | --- | --- | --- | --- | --- | --- | --- | --- | --- |
| DAVID | Fishers | Yes | | No | | No | | Updated November 2023 | Updated May 2023 | Bonferroni, Benjamini-Hochberg FDR, Two-stage Benjamini-Hochberg FDR | Yes | No | Yes |
| gProfiler | Fishers | Yes | | Yes | | Yes | | Updated 01-22-2024 | Updated January 2024 | g:SCS threshold, Bonferroni, Benjamini-Hochberg FDR | Yes | No | No |
| FishEnrichr | Fishers | Yes | | No | | Yes | | KEGG 2019 | GO 2018 | Benjamini-Hochberg FDR | Yes | No | No |
| GSEApy | Fishers | No | | Yes | | No | | KEGG 2019 | GO 2018 | Benjamini-Hochberg FDR | No | No | No |
| LRPath | Logistic Regress-ion | Yes | | No | | Yes | | Bioconductor KEGG.db (2011) | Bioconductor KEGG.db (2011) | FDR | No | No | No |
| clusterProfiler | Fishers | No | | No | | Yes | | Bioconductor KEGG.db (2011) or option for latest version using API | Bioconductor KEGG.db (2011) | Benjamini-Hochberg FDR | Yes | No | No |
| GOAtools | Fishers | No | | Yes | | No | | No KEGG support | Latest Version (updates automatically) | Bonferroni, Benjamini-Hochberg FDR | No | No | No |
| danRerLib | Fishers and Logistic Regress-ion | No | | Yes | | No | | Latest Version (updates automatically) | Latest Version (updates automatically) | Bonferroni, Benjamini-Hochberg FDR | Yes | Yes | Yes |

**Table S2**: KEGG functional enrichment analysis testing comparisons. The number of included KEGG pathways is the number of zebrafish annotated pathways available to test against while the danRerLib orthology enrichment tool includes zebrafish annotated pathways when they exist and those mapped to zebrafish from human annotated pathways.

| Enrichment Tool | Number of Included KEGG Pathways | Number of Enriched KEGG Pathways | Number of Depleted KEGG Pathways | Number of Upregulated KEGG Pathways | Number of Downregulated KEGG Pathways |
| --- | --- | --- | --- | --- | --- |
| DAVID | 178 | 10 | N/a | 1 | 15 |
| gProfiler | 179 | 7 | 0 | 1 | 6 |
| FishEnrichr | 151 | 14 | N/a | 5 | 14 |
| GSEApy | 151 | 14 | N/a | 5 | 14 |
| clusterProfiler | 179 | 17 | N/a | 7 | 20 |
| LRPath | 155 | 17 | 12 | 25 | 13 |
| danRerLib enrich_fishers | 179 | 17 | 3 | 7 | 20 |
| danRerLib enrich_logistic | 179 | 16 | 17 | 29 | 26 |
| danRerLib enrich_fishers orthology | 357 | 26 | 14 | 17 | 27 |
| danRerLib enrich_logistic orthology | 357 | 24 | 50 | 60 | 35 |

**Supplementary Figures**

**Figure S1**: Enrichplots module additional plotting options for the data presented in Figure 1. (A) Bar chart displaying the top 10 downregulated pathways. (B) Volcano plot displaying the relationship between the odds ratio and the p-value for upregulated and downregulated pathways.

**Supplementary References**

Sherman, B.T., et. al. (2022). DAVID: a web server for functional enrichment analysis and functional annotation of gene lists (2021 update*). Nucleic Acids Research*, *50*(W1), W216–W221.

Huang, D.W., Sherman, B.T., & Lempicki, R.A. (2009). Systematic and integrative analysis of large gene lists using DAVID Bioinformatics Resources. *Nature Protocols*, 4(1), 44-57.

Kolberg, L., et. al. (2023). g:Profiler—interoperable web service for functional enrichment analysis and gene identifier mapping (2023 update). *Nucleic Acids Research*, *51*(W1), W207–W212.

Chen, E.Y., et. al. (2013). Enrichr: interactive and collaborative HTML5 gene list enrichment analysis tool. *BMC Bioinformatics*, 14(128).

Kuleshov, M.V., et. al. (2016). Enrichr: a comprehensive gene set enrichment analysis web server 2016 update. *Nucleic Acids Research*, *44*(W1), W90–W97.

Fang, Z., Liu, X., Peltz, G. (2022). GSEApy: a comprehensive package for performing gene set enrichment analysis in Python. *Bioinformatics*, *39(*1), btac757.

Sartor, M.A., Leikauf, G.D., & Medvedovic, M. (2009). LRpath: a logistic regression approach for identifying enriched biological groups in gene expression data. *Bioinformatics*, *25*(2), 211–217.

Wu, T., et. al. (2021). clusterProfiler 4.0: A universal enrichment tool for interpreting omics data. *The Innovation*, *2*(3), 100141.

Yu, G., Wang, L., Han, Y., & He, Q. (2012). clusterProfiler: an R package for comparing biological themes among gene clusters. *OMICS: A Journal of Integrative Biology*, *16*(5), 284-287.

Klopfenstein, D.V., et. al. (2018). GOATOOLS: A Python library for Gene Ontology analyses. *Scientific Reports*, *8*(10872).

Pan, K.H., Lih, C.J., & Cohen, S.N. (2005). Effects of threshold choice on biological conclusions reached during analysis of gene expression by DNA microarrays. *Proc Natl Acad Sci U S A*, *102*(25), 8961-8965.
